# Early markers of the metabolic syndrome in children born post-term

**DOI:** 10.1186/1687-9856-2013-S1-O22

**Published:** 2013-10-03

**Authors:** Ahila Ayyavoo, Paul L Hofman, José GB Derraik, Sarah Mathai, Peter Stone, Lynn Sadler, Wayne S Cutfield

**Affiliations:** 1 Liggins Institute, University of Auckland, Auckland, New Zealand; 2 National Research Centre for Growth and Development, University of Auckland, Auckland, New Zealand; 3 Department of Obstetrics and Gynaecology, Faculty of Medical and Health Sciences, University of Auckland, Auckland, New Zealand; 4 National Women’s Health, Auckland District Health Board, Auckland, New Zealand

## 

We recently showed from a Swedish cohort that nearly half of boys born post-term (≥42 weeks gestation) were overweight or obese at 16 years of age. We hypothesized that post-term children would display features of insulin resistance and the metabolic syndrome even in their pre-pubertal years.

90 healthy pre-pubertal children aged 4–11 years, with birth weight appropriate-for-gestational-age were studied: 36 children born post-term (18 boys and 18 girls) and 54 children (36 boys and 18 girls) born at term (38–40 weeks). Insulin sensitivity was measured using Bergman’s minimal model. Other assessments included fasting lipid and hormonal profiles, body composition using whole-body dual-energy x-ray absorptiometry, and 24-hour ambulatory blood pressure monitoring.

Insulin sensitivity was reduced in post-term children (8.44±0.74 vs 13.55±0.89 x10^-4^·min^−1^·(mU/L); p<0.0001). Post-term children had an adverse lipid profile, with higher total cholesterol (4.26±0.17 vs 3.92±0.11 mmol/l; p=0.023) and LDL (2.51±0.13 vs 2.25±0.08 mmol/l; p=0.016) concentrations. Further changes suggestive of the metabolic syndrome among post-term children included an increased android to gynoid fat ratio (0.74±0.03 vs 0.61±0.02; p=0.026), and a reduction in the normal nocturnal systolic blood pressure dip (8.2±1.0 vs 13.8±1.0%; p=0.016). Post-term children also had higher serum leptin (7.18±0.91 vs 3.67±0.41 ng/ml; p=0.011), lower adiponectin (8226±693 vs 10536±556 ng/ml; p=0.046), and lower IGFBP1 (9.56±1.06 vs 18.03±1.59 ng/ml; p=0.032) concentrations.

Our study shows for the first time that post-term children have early features of the metabolic syndrome, including reduced insulin sensitivity, adverse lipid profile, and increased abdominal adiposity.

